# A Review of Common Cyanotoxins and Their Effects on Fish

**DOI:** 10.3390/toxics11020118

**Published:** 2023-01-25

**Authors:** Halina Falfushynska, Nadiia Kasianchuk, Eduard Siemens, Eliana Henao, Piotr Rzymski

**Affiliations:** 1 Department of Marine Biology, Institute for Biological Sciences, University of Rostock, 18059 Rostock, Germany; 2 Faculty of Electrical, Mechanical and Industrial Engineering, Anhalt University for Applied Sciences, 06366 Köthen, Germany; 3 Faculty of Biology, Adam Mickiewicz University, 61712 Poznan, Poland; 4 Research Group Integrated Management of Ecosystems and Biodiversity XIUÂ, School of Biological Sciences, Universidad Pedagógica y Tecnológica de Colombia, Tunja 150003, Colombia; 5 Department of Environmental Medicine, Poznan University of Medical Sciences, 61701 Poznan, Poland; 6 Integrated Science Association (ISA), Universal Scientific Education and Research Network (USERN), 61701 Poznań, Poland

**Keywords:** cyanobacteria, ecotoxicology, toxin bioaccumulation, aquatic environment

## Abstract

Global warming and human-induced eutrophication drive the occurrence of various cyanotoxins in aquatic environments. These metabolites reveal diversified mechanisms of action, encompassing cyto-, neuro-, hepato-, nephro-, and neurotoxicity, and pose a threat to aquatic biota and human health. In the present paper, we review data on the occurrence of the most studied cyanotoxins, microcystins, nodularins, cylindrospermopsin, anatoxins, and saxitoxins, in the aquatic environment, as well as their potential bioaccumulation and toxicity in fish. Microcystins are the most studied among all known cyanotoxins, although other toxic cyanobacterial metabolites are also commonly identified in aquatic environments and can reveal high toxicity in fish. Except for primary toxicity signs, cyanotoxins adversely affect the antioxidant system and anti-/pro-oxidant balance. Cyanotoxins also negatively impact the mitochondrial and endoplasmic reticulum by increasing intracellular reactive oxygen species. Furthermore, fish exposed to microcystins and cylindrospermopsin exhibit various immunomodulatory, inflammatory, and endocrine responses. Even though cyanotoxins exert a complex pressure on fish, numerous aspects are yet to be the subject of in-depth investigation. Metabolites other than microcystins should be studied more thoroughly to understand the long-term effects in fish and provide a robust background for monitoring and management actions.

## 1. Introduction

Global warming and human-driven eutrophication accelerate the occurrence of cyanobacteria in surface waters by means of establishing optimal conditions for them to thrive [1]. Higher temperatures and nitrogen and phosphorus availability stimulate the growth of these microorganisms and can eventually lead to their massive blooms [2,3]. According to the CyanoMetDB database, over 2000 cyanobacterial metabolites have been identified, including, among others, microcystins, cyanopeptolins, other depsipeptides, anabaenopeptins, microginins, aeruginosins, cyclamides, cryptophycins, STXs, spumigins, microviridins, and anatoxins [4]. The majority of cyanotoxins are released into the water column only following cyanobacterial death, although some can also be actively released by the intact cells. They reveal diversified mechanisms of action, encompassing cyto-, neuro-, hepato-, nephro-, and neurotoxicity. The toxicity of various cyanobacterial metabolites remains yet to be studied [5,6]. The most studied metabolites produced by cyanobacteria include hepatotoxic microcystins (MCs) and nodularins (NODs), neurotoxic anatoxin-a (ATX-a), saxitoxins (STX) and beta-methylamino-L-alanine (BMAA), cytotoxic cylindrospermopsin (CYN), and dermatotoxic lyngbyatoxins and aplysiatoxins [2,5].

Among these, MCs are the most commonly studied [2]. Due to the different compositions of the two variable protein amino acids, over 100 MC variants have been discovered [7]. The most common MCs include MC-LR, MC-RR, MC-YR, and MC-LA (L, leucine; R, arginine; Y, tyrosine; and A, alanine) [7,8]. From these, MC-LR is the most investigated and potently toxic. Along with NODs, MCs are cyclic heptapeptides and pentapeptides, characterized as having the unusual β-amino acid Adda (3-amino-9-methoxy-2, 6, 8-trimethyl-10-phenyldeca-4E, 6E dienoic acid), which is usually linked to their severe toxicity [7,8,9]. In vertebrates, they act as the inhibitors of serine/threonine-specific protein phosphatases 1 and 2A (PP1 and PP2A), causing cytolysis with subsequent apoptosis of hepatocytes [10,11]. MCs are synthesized by many cyanobacteria species, such as representatives of the *Microcystis* (most commonly by *Microcystis aeruginosa*), *Oscillatoria*, *Aphanizomenon*, *Anabaena*, *Planktothrix,* and *Anabaenopsis* genera, while NOD production was identified in *Nostoc* genera, *Nodularia spumigena* Mertens ex Bornet & Flahault, and *Nodularia sphaerocarpa* Bornet & Flahault [5,6,8,12,13].

ATX-a and STXs are much less distributed, although they reveal high toxicity in vertebrates. ATX-aa, the most prominent member of the group, is produced by representatives of *Anabaena*, *Planktothrix,* and *Aphanizomenon* genera [14,15]. ATX-a functions as an irreversible active site-directed inhibitor of acetylcholinesterase that alters muscle junctions. Its effects range from muscle weakness or fasciculations to paralysis and even death due to respiratory failure. Some other variants of anatoxins include homoATX-a (hATX-a), dihydroATX-a, dihydrohomoATX-a, and ATX-a(S) (suggested by some authors to be renamed as guanitoxin to emphasize its distinctive guanidino organophosphate chemical structure [16]). Although the chemical structure may differ, their modus operandi is quite similar, and so are the consequences they entail. All STXs share a unique chemical structure characterized by a tricyclic backbone with diverse side chains attached thereto. Exposure to STXs may lead to cardiac arrhythmias since these compounds block calcium and potassium channels [17]. In acute cases, STXs paralyze respiratory muscles and cause death due to the selective inhibition of sodium channels [2]. CYN is a cyanobacterial cytotoxin produced by various cyanobacteria belonging to *Raphidiopsis*, *Chrysosporum,* and *Aphanizomenon* genera. In mammals, CYN primarily targets the liver, though it also impairs the kidney, lungs, spleen, and nervous system by inhibiting protein biosynthesis, followed by cytotoxicity and cell death [15,18].

In the present paper, we review data on the occurrence of the most studied cyanotoxins, MCs, NODs, CYN, ATX-A, and STXs, in the aquatic environment, as well as their potential bioaccumulation and toxicity.

## 2. Spatial Distribution of Common Cyanotoxins in Water Bodies

Even though cyanobacteria are spread worldwide, understanding the geographical distribution of cyanotoxins can be, in some instances, a challenging task due to the unbalanced allocation of the obtained data and differences in research efforts. However, MCs are the most commonly identified cyanotoxins worldwide and can be present in surface waters in Europe, North and South America, Oceania, and Africa [12,19,20,21,22,23,24,25,26,27,28,29,30,31].

Among all Asian countries, China has the highest number of papers focusing on cyanotoxins; however, in terms of geographical distribution, the studies are mainly concentrated on specific areas of the country. Most of the research was conducted in the middle and lower reaches of the Yangtze River and the Yunnan–Guizhou Plateau [32]. *Microcystis* comprises the majority of cyanobacteria in China’s water bodies [33], followed by *Dolichospermum*/*Aphanizomenon* complex, *Raphidiopsis*, and *Planktothrix*/*Planktothricoides* [34]. The cyanotoxin concentrations differ across various water bodies in China, with high concentrations found in, e.g., Lake Taihu, leading to the water crisis in its basin with temporary interruptions in access to drinking water [35,36,37].

Other Asian countries also documented cyanobacteria contamination. For instance, in Japan, more than one-third of all microbial strains listed in the National Institute of Environmental Studies of Japan collection belong to the *Microcystis* genus [38]. Interestingly, although *Microcystis*, *Dolichospermum*, and *Planktothrix* were reported in the water bodies of western Japan, only the first genus was evidenced to produce the MC [39]. Other cyanotoxins were also found in Japan but at considerably lower concentrations [40,41].

According to reports, Chinese and Canadian water bodies have the highest levels of MCs, which are often much higher than the World Health Organization’s permissible level of 1 μg/L, which has been mandated in many countries. In detail, the detection frequency of MC-LR in drinking water sources in several river basins in China was 55.5%, and its concentration ranged from 0.06 × 10^−3^ to 52 × 10^−3^ μg/L, with an average value of 12.5 × 10^−3^ μg/L [27]. Likewise, the highest mean concentration of MCs in lakes in Canada and the USA was 2.5 μg/L and 16 μg/L, respectively, but in some cases, reached 11 μg/L and 86 μg/L [29,30]. Moreover, MCs were detected in raw water samples from Tendaha Lake (Saudi Arabia) in concentrations ranging from 2 to 8 μg/L [26].

Several cases of MC poisoning were also recorded on the continent: dozens of wild and domestic animals had unusual illnesses following the death in lake areas of the Rift Valley south of Addis Ababa [42] and Kruger National Park in South Africa [43]. The recent death of more than 330 African elephants (*Loxodonta africana* (Blumenbach)) in Botswana was suggested to be potentially associated with toxic cyanobacteria bloom [44]. To this end, the dynamic of algal blooms and MC-produced cyanobacteria all over the world is a matter of concern and the continuous and profound control of MCs, therefore, is in demand.

CYN is Europe’s second-most-researched cyanotoxin [12,16]. In Europe, CYN-producing cyanobacteria were often researched in Poland and Germany [12,45,46] but were also found in France, Italy, Spain, Greece, Sweden, Portugal, Hungary, Ukraine, and the Czech Republic [16,23,47,48,49,50]. The median concentration of CYN is reported to be the highest in the Asia/Pacific and South America regions, with values as high as 2.3 μg/L [51], which exceeds the permissible value by more than four-times, followed by Europe (0.50 μg/L) and North America (0.35 μg/L). However, an extreme case of CYN contamination was found in Italy, with a concentration as high as 126 μg/L [52]. Unfortunately, the information regarding CYN is limited when compared to MCs [51]. Nevertheless, CYN is reported to be frequent worldwide [53,54]. Due to the high cost of analytic procedures and the challenges involved in cultivating, collecting, and preparing cells for analysis, cyanobacterial toxins such as CYN are not routinely monitored in all regions of the world [51]. Meanwhile, relatively recent events of the expansion of potential CYN-producing cyanobacteria strains, e.g., *Raphidiopsis raciborskii* (Woloszynska) Aguilera & al., were recorded in European and Chinese cold waters due to global warming and increasing eutrophication [23,55]. However, no *R*. *raciborskii* strain identified in European surface waters was documented to produce CYN. This species was also identified in South America, including Brazilian water reservoirs [56]. A molecular study revealed that although Brazilian strains can possess a CYN synthetase cluster with the complete *cyrA* sequence and parts of *cyrB* and *cyrC,* they cannot produce the toxin due to absence of the *cyrJ* gene. Interestingly though, they are capable of producing STXs [57]. North American and African strains were also not implicated in CYN production [16]. Nevertheless, there is a need to continue global CYN monitoring, identify its producers, and mitigate its occurrence and toxicity to biota.

## 3. Factors Controlling Cyanotoxin Production

Cyanotoxins are spread worldwide, both in aquatic and terrestrial habitats, constituting an indispensable part of global biodiversity, food chains, and biogeochemical cycles. A set of geological, environmental, and anthropogenic factors can influence their natural state. Usually, the formation of such blooms requires increased temperature, high availability of photosynthetically active radiation, windless weather, and pH in a 6–9 range [58]. Wang et al. [44] recorded the climate conditions in places where acute poisonings and mass mortalities of wild animals occurred. The results showed that dry and hot weather stimulates the proliferation of cyanotoxins, which considerably increases the risk of poisoning. The temperature has been shown as a primary factor explaining the occurrence of MCs in European surface waters [59]. Furthermore, it has been demonstrated that an increase in temperature by 10 °C resulted in the overexpression of *mcyB* in *M. aeruginosa* SAG14.85 [60]. One should note that toxigenic cyanobacteria are also increasingly observed to occur and form blooms during the winter period [61].

Noteworthily, cyanotoxin production does not always correlate with the abundance of cyanobacteria in water bodies [62]. For instance, Walls et al. [63] showed a negative correlation between the biomass of *Planktothrix agardhii* (Gomont) Anagnostidis & Komárek and MC production (>19 °C), meaning that the highest MC release occurs under unfavorable conditions for algae. Plausibly, the primary explanation for such phenomena is that cyanotoxins are secondary metabolites of cyanobacteria, and their release may have a signaling or protectant role.

The other critical factor needed for producing cyanotoxins is the presence of nutrients. Under nitrogen deficiency, MC concentration in *M*. *aeruginosa* decreases, with a possible further increase when N becomes available again [64]. However, no strong evidence exists that nutrient enrichment regulates gene expression and biosynthesis of MCs. One study investigated that high nitrogen levels positively influenced the MC synthesis of *Microcystis* [65], while others reported a similar conclusion for *M*. *aeruginosa* under nitrogen starvation. The changes in the nutrient composition and N:P ratio showed no influence on the proportion of MC variants and the total amount of toxins [66]. At the same time, the combination of elevated temperatures and high nitrogen concentrations boosts the proliferation of cyanotoxins [67]. Simultaneous enhancement of the abovementioned factors entailed the significant growth of MC synthesis compared to control and other treatment types [68].

Pesticide pollution can also alter the synthesis and toxicity of cyanotoxins [69]. Both lindane and glufosinate upregulated the expression of genes involved in MC biosynthesis (namely *mcyA* and *mcyD*), and glyphosate was shown to induce the extracellular release of MCs [70,71,72]. Furthermore, carbaryl magnifies the adverse effect of MC on *Daphnia pulicaria* Forbes, showing their synergic activities [73]. Other common anthropogenic pollutants are heavy metals that are especially widespread in industrial and agricultural territories. Iron, copper, zinc, and manganese ions induce cyanobacterial growth and toxin biosynthesis, both when the concentrations of such metals are elevated and limited [74]. Such a result was corroborated by other studies, as adding Cu, Mo, or Co boosted cyanobacterial growth and productivity by up to 40% [75]. Therefore, the synthesis of cyanotoxins is based upon the multiplicity of factors subject to further research, as it will provide the necessary insights into understanding how to normalize the number of cyanotoxins found in our ecosystem.

## 4. Bioaccumulation of Cyanotoxins in Fish

Fish consumption is a vital part of the world food industry. The State of World Fisheries and Aquaculture [76] estimated that fish constitutes 17% of world animal proteins and 7% of all types of protein intake. In several countries, such as Bangladesh, Cambodia, the Gambia, and Ghana, this number rises to approximately 50%. It has been demonstrated that consuming fish is a major and, in some cases, the only route through which humans are exposed to MC, and that doing so can result in MC intakes that exceed the safety levels [17,77,78]. Therefore, understanding the accumulation of cyanotoxins by fish is critical in preventing any potential human poisonings.

MC accumulation is the most widely studied among all types of cyanotoxins. Its bioaccumulation potential is generally lower in fish compared to zooplankton or other invertebrates [79]. Moreover, planktivorous fish accumulate a higher average amount of cyanotoxins than carnivorous fish. For example, in planktivorous fish, the highest concentrations of MC reached over 874 µg/g dw, while the maximum reported concentration for carnivores reached only ≈ 50µg MC/g in *Perca fluviatilis* L. [80]. MC absorption in carnivores is likely to occur via an alternative route, namely the gills, but in planktivores and omnivores, MC transport into internal organs is essentially constrained to movement across the gastrointestinal wall [81]. MCs accumulated rapidly in rainbow trout, yellow perch, and lake trout, reaching up to 11 ng/g of liver tissue. The process is completed within 2–6 h of exposure [82,83]. Since 1% of the MC-LR administered dose can accumulate in animal liver tissue [84], water animals that are regularly exposed to algal blooms are at risk of being exposed to cyanotoxins, which can have an impact on their health and population biodiversity.

In general, the liver is indicated to be the primary target organ for MC accumulation, followed by other blood-irrigated organs [83,85,86,87], which is associated with the presence of the unspecific anionic transporters localized on the hepatocytes [88]. However, it was not always the case that the highest concentrations of MCs were accumulated in the liver: in a number of cases, a higher amount was shown in the intestines. MC presence was also found in the fish brain, meaning it can probably cross the blood–brain barrier [85,89].

Hepatocytes rapidly absorb MC via carrier-mediated transport, which relies on OATP1B/Oatp1b isoforms [88]. Mice lacking Oatp1b2 are able to transfer less MC into the liver and show reduced liver damage [90]. In zebrafish and rainbow trout, Oatp transporters have been found to play a significant role in MC transport into cells and its accumulation so far [91,92]. The affinity and capacity of transporting MC of zfOatp1d1, which is expressed predominantly in the liver and brain with about 30–50-fold lower expression in skeletal muscle, gills, and intestine, determined the toxicological impact of cyanobacterial blooms [92].

Saker and Eaglesham [93] were the first to report CYN bioaccumulation in fish from a water column containing ≈590 μg/L of the studied toxin. They reported 4 μg/g/of CYN in freeze-dried hepatopancreatic tissues and 9 μg/g in freeze-dried muscle tissue in *Cherax quadricarinatus* (von Martens). The bioaccumulation potential of this toxin in aquatic species has been indicated by research that proposes a bioaccumulation factor of CYN up to 171 [51], and fish ingestion of cyanobacterial cells as the potential route of exposure has also been nominated. However, data and mechanistic conceptions regarding CYN accumulation in fish are generally strictly limited, which defines an insufficient analysis of the issue.

Some studies showed that only a small fraction of a given dose of MCs was eliminated through urine and feces. To recap, Robinson et al. [94] found that the liver accumulated 83% of the entire dosage and that 24% of the radiolabeled MC-LR was found in urine (9%) and feces (15%). The most obvious form of MC-LR detoxification is the conjugate MC-LR-GSH, which was reported to be less toxic than the initial MCs. For instance, the MC-LR-GSH metabolite inhibition potential against protein phosphatase is reduced by 3–10-times when compared to pure MC-LR [95]. Although MC-LR-GSH is believed to be less toxic, almost all studies have been conducted on murine models. More research is needed to understand the metabolism of MCs and other cyanotoxins in fish and to estimate how toxic the metabolites of common cyanotoxins are.

## 5. Oxidative Stress Is the Dominant Downstream Adverse Effect of Cyanotoxins

As mentioned above, fish tend to bioaccumulate cyanotoxins, which eventually manifest in numerous adverse effects, including hepatotoxicity, neurotoxicity, oxidative stress, immune dysfunction, and reproductive toxicity.

Aside from specific toxicity signs, such as protein phosphatase (PP1 and PP2A) inhibition by MCs, cyanotoxin uptake can result in acute poisonings in invertebrate and vertebrate animals, as well as humans, resulting in significant tissue damage and deteriorating health status. Such severe effects can be caused by antioxidant system alterations and reactive oxygen species production.

In recent decades, a growing body of research has suggested that MCs can affect the antioxidant system and/or cause oxidative stress in a wide range of aquatic animals. Antioxidant defense system attenuation, lipid peroxidation, DNA damage, and mitochondrial and endoplasmic reticulum damage have all been linked to the absorption of MCs [54,96,97]. Although these disorders have been disclosed, the underlying processes and mechanisms have received less attention. Similar state-of-the-art links exist to other toxins than MCs, namely CYN or anatoxin. If some information regarding CYN and anatoxin toxicity in murine models is known, data regarding their effects and mechanisms of action on aquatic biota, which are exposed to cyanotoxins instantly, are strictly limited.

MC, the most common type of cyanotoxin, is proven to increase ROS levels in living organisms. Li et al. [98] showed an almost two-fold induction of the ROS level in common carp hepatocytes after 6 h of exposure to 10 μg/L MC-LR. Likewise, a similar increment in hydroxyl radical (∙OH) content was ascertained for *Cyprinus carpio* L. exposed to 10 μg/L MC-LR for 12 h or 50–120 μg/kg, with subsequent production diminution over time [99,100]. ROS, which is tightly related to mitochondrial membrane integrity and apoptosis, plays a crucial role as the second messenger in the MC toxicity pathways. As is evident, MC-LR induced ROS overproduction in primary mouse hepatocytes by dysregulating the expression and activity of the pro-oxidants SOD1, MAOA, and NOX4 and the antioxidant GPX1, and subsequently causing plasma membrane rupture, diffusion of the PI-stained nuclei, as well as upregulation of the expression of necroptotic and apoptotic proteins [101] (ROS was shown to be elevated following exposure to MC-LR in *C. carpio* and agreed with overexpression of apoptosis-related genes, including p38, JNKa, and bcl-2 [100]). Likewise, ROS production in *C. carpio* hepatocytes stimulated by crude extracts of European strains of *R. raciborskii* went hand in hand with oxidative lesion accumulation, caspase-3 activation, and DNA damage [102].

Lipid peroxidation (LPO) is the most studied consequence of oxidative damage in biological systems because it reflects the loss of cellular function in response to oxidative stress and the disruptive actions of ROS, as well as being proven to be a non-specific marker of the harmful effects of xenobiotics on biota. Changes in cell membrane structure related to phospholipids cause disruptions in permeability, ionic flux, and DNA modifications, which eventually lead to cell death [103]. Similarly, *Oreochromis niloticus* L. exposed to MC-LR showed a significant increase in LPO in the liver [104]. Accumulation of the TBA-reactive products and protein carbonyls was also reported in zebrafish exposed to pure cytotoxins MC-LR and CYN and cyanobacterial extracts produced from *Aphanizomenon gracile* Lemmermann and *R*. *raciborskii* [54]. Furthermore, crude extracts of European strains of *R. raciborskii* (not producing CYN) and CYN analogues promote significant elevations in lipid peroxidation and protein carbonylation in carp hepatocytes [105]. However, when *Tinca tinca* L. was orally exposed to cyanobacterial extracts (5–55 mg MC-LR/fish), there was an increase in brain LPO but no changes in protein peroxidation [106]. The fact that LPO has been found to appear in the presence of a complete lack of protein oxidation suggests that LPO is a more sensitive sign of cyanotoxic effects on fish than protein carbonylation. Moreover, the correspondence between LPO and ROS in the liver tissue of fish that was proved to exist allows for the determination of LPO as the early sign of membrane destabilization and the possible development of mitochondrial and endoplasmic reticulum stress, which can result in apoptosis and necrosis.

Substantial changes were observed in glutathione (GSH) level and glutathione S-transferase (GST) activity. In common carp, GSH level decreased tremendously after only 15 min under 10 μg/L MC-LR, followed by hepatocytes exhibiting depletion of GSH after 6 h exposure [98]. The results were corroborated by several other studies [11,99,107]. However, it is complicated to find a general drift of GSH response to MCs, as, e.g., Sun et al. [108] showed increased levels of GSH, while some others indicated no meaningful difference at all [10,11,109].

By and large, the MC effect on GST activity varied among studies, having no evident general trend [110]. Wang et al. [111] indicated an approximately 80% increase in sGST mRNA expression after 24 h of MC-LR influence, while Fu and Xie [112] showed elevated changes in expression over time but with an apparent decrease in most GST mRNAs at 12–24 h of exposure. Despite being somewhat different, changes in GST activity were shown in various aquatic organisms, including fishes [99,113,114,115,116,117]. In addition, MC-LR induced a considerable increase in GST activity in the liver but a reduction in gills in the same *Brycon amazonicus* (Spix & Agassi) specimens [11]. Apparently, sGST contributes to MC detoxification in fish by forming a specific MC-LR glutathione conjugate [118]. Conjugation of MC-LR with cysteine and GSH led to the formation of 3–10-times less toxic compounds than MCs alone [95]. It provides further evidence that the MC conjugation catalyzed by GST is the initial stage in the detoxification process and corroborates with our findings in zebrafish when GST was significantly depleted by 20 µg/L MC-LR and 20 µg/L CYN exposure for 14 days, resulting in signs of cytotoxicity, such as LDH leakage and RAD51 expression variation, which takes part in DNA damage repair [54]. Furthermore, CYN induced RAD51 downregulation while MC-LR retained the ability to stimulate it, supporting the idea that CYN is more genotoxic than MC-LR, as discovered in human hepatoma cells HepG2 [53].

Ever since its discovery and subsequent significance in the antioxidant response, Nrf2 has been hailed as a positive transcription factor that plays a pivotal role in supporting organisms to tackle the consequences of oxidative stress and the toxic effects of some metals and organic pollutants. The Nrf2 pathway can be activated in two ways, both of which have been discovered in mammals. Some substances have been shown to activate the Nrf2 pathway by disrupting the Nrf2/Keap1 complex in the nucleus, while others allow Nrf2 to escape Keap1-dependent degradation, leading to stabilization of Nrf2, increased nuclear localization of Nrf2, and activation of Nrf2 [119,120]. Based on studies in human cell lines, it has been proposed that MC-LR likely activates Nrf2 by binding to the cytosolic regulator Keap1 proteins and releasing Nrf2 [121]. The results were corroborated by several other studies [122]. Moreover, HepG2 and Hep3B cell proliferation is stimulated by MC-LR-induced overexpression of Nrf2, suggesting a stimulative function for Nrf2 in carcinogenesis [121]. However, the possible modulation of Nrf2 by MCs is a scarcely studied issue in fish and other water animals. To elaborate, zebrafish exposed to MC-LR and CYN and bioactive compounds extracted from cyanobacterial strains showed decreased transcript levels of Nrf2 [54]. On the other hand, the expression levels of CpNrf2 were raised in hepatopancreas of the freshwater mollusk *Cristaria plicata* (Leach) after injection of 0.2 mg MCs but decreased in the case of co-exposure with siRNA 323 [123]. To this end, the abovementioned findings suggested that MCs might as well stimulate Nrf2, which finally may raise adaptive mechanisms in response to oxidative stress, but deep diving into the mechanisms of Nrf2 involvement in adaptive responses to cyanotoxin-induced stress is highly appreciated.

## 6. Immunomodulatory and Inflammatory Effects of Cyanotoxins

Cyanotoxins, including MCs and CYN, have been studied for their ability to modulate interleukin-1 and TNF, stimulate iNOS in macrophages, and cause degeneration and necrosis of cortical lymphocytes in the thymus as well as lymphophagocytosis in mice used as experimental models [124,125,126]. Although they have immunotoxicity potential, their effects on immune-related parameters in fish have not been properly investigated. MCs have been recognized, meanwhile, as the most studied. Goldfish Carassius auratus L. fed MC-rich diets (20% and 40% of 1.41 mg/g MC dry weight) demonstrated a significant increase in lysozyme activity in the low-exposed group but a significant decrease in the higher-exposed group [127]. In another study, blunt snout bream (Megalobrama amblycephala Yih) were fed diets containing 15% and 30% lyophilized powder of a mixture of MC-LR and MC-RR for 30 days [128]. MCs were accumulated in all tissues by blunt snout breath, with the kidney and liver being the most prone to accumulate them while muscle and heart only barely absorbed toxins. The exposure also resulted in increased lymphocyte peripheral interspace, nucleus shrivel, and edematous mitochondria of head kidney lymphocytes, as well as decreased macrophage phagocytosis activity. Further, the MC-enriched diet downregulated the mIgD and sIgZ immunoglobulin genes in the head, kidney, and spleen of M. amblycephala when the response of sIgM was dose responsive, with a significant decrease in the high-dietary-toxicity group [128]. The stimulation of immunoglobulin (IgA, IgM, and IgG) production and oppression of antimicrobial peptides (brevinin-1PLc, brevinin-2GHc, and ranatuerin-2PLa) and lysozyme as a function of exposure concentrations was also noted in higher vertebrates, i.e., tadpoles of Lithobates catesbeianus (Shaw) exposed to 0.5, and 2 μg/L of MC-leucine arginine (MC-LR) for 30 days [129].

Phagocytosis has been recognized as an important mechanism of the innate immune system in fish. The review of existing data indicates that there is no standard reaction of phagocytic activity in fish exposed to cyanotoxins. The reaction is reported to be concentration- and species-dependent, and it varies from profound suppression to significant upregulation. Some findings emphasized that phagocytosis activity increases significantly after low exposure to cyanotoxins, namely MC, and decreases significantly when cyanobacteria concentrations in the environment increase [128]. Some others showed that only high concentrations of cyanotoxins are able to induce phagocytosis. As an example, the phagocytic ability and respiratory burst activity were elevated in the cells of rainbow trout in the presence of MC-LR at a dose of 5 μg/mL, whereas in the case of low exposure, the phagocytic ability remained unwavering [130]. Lastly, some citations claim cyanotoxins, dominantly MC, as the repressor of phagocytic activity [131], which was described, in particular, in carp embryos and larvae exposed to 1.3 µg/L or 13 µg/L MCs [132]. Although phagocytosis is vulnerable to cyanotoxins, the mechanism of the observed changes remains unknown. Obviously, it may interact with phosphorylation and dephosphorylation processes, such as tyrosine phosphorylation cascades, which aim to initiate and terminate signals, leading to cell migration, infection clearance [133], as well as cytokine production [134]. We have recently shown that a stress-responsive Ser/Thr protein kinase MK2, which plays a crucial role in p38 MAP kinase-dependent cellular stress signaling, regulating cytokine production and inflammation, was transcriptionally downregulated in the liver of zebrafish Danio rerio (Hamilton) exposed to purified MC-LR and CYN or to the extract of A. gracile SAG 31.79 [54].

In general, low doses and concentrations of cyanotoxins, including MCs, in diets or media may be considered immune provoking for fish, whereas acute or even high concentrations are immunosuppressive. The same trend was revealed in higher vertebrates, i.e., the L. catesbeianus [129] and human cells [135]. In response to secondary infection, exposure to cyanotoxins is likely to increase the likelihood of pathogenic bacterial infestation while also prompting the organism to strengthen other relevant immunological responses, and some findings support our hypothesis. In particular, MC-LR exposure provoked more intensive cumulative mortality in Macrobrachium rosenbergii De Man infected with Vibrio vulnificus (Reichelt) Farmer and Aeromonas hydrophila (Chester) Stanier than in the corresponding control group in a time–dose-dependent manner. The latest event agreed with the prominent suppression of the expression of the immune-related genes, namely crustin 1, crustin 3, lysozyme-i, proPO, ALF1, and hemocyanin, as well as inhibition of alkaline phosphatase [136].

While it is clear that cytokines, such as TNF and IL1, as well as the TLR/MyD88 signaling pathway, can control the immune defense against cyanotoxins in higher vertebrates, the mechanism for this phenomenon in fish is still unknown. Some research has been conducted recently on inflammation induced predominantly by MCs. Short-term exposure of carp head kidney and blood leukocytes to 0.01 or 0.1 μg/mL MC-LR and ATX-a altered the expression of pro-inflammatory (IL-1β and TNF-α) and anti-inflammatory (TGF-β and IL-10) cytokines. Expression of the cytokine IL-1β was highly upregulated following Antx-α, which exerted a more profound effect than MC-LR [137]. Similar findings were presented in zebrafish after animal treatment with 0.4–10 μg/L MC-LR for 60 days [138] and in mice, both in vivo in animals orally exposed to MC-LR (1–30 μg/L for 180 days) and in vitro in cultured Sertoli cells [139,140]. Furthermore, secreted TNF-α could bind to TNFR1, cleave caspase-8, activate caspase-3, and induce apoptosis in a time-dependent manner [139]. A few references, on the other hand, support the idea that MCs act as a pro-inflammatory cytokine suppressor and downregulate TNF-α, type I IFN, IL-1β (only in the kidney), and PGRP-L genes in the spleen and head kidney of grass carp intraperitoneal injection with 50 μg MC-LR/kg [141]. However, the downregulation of pro-inflammatory cytokines could not negate the total inhibition of immune function in exposed fish [141]. To this end, cyanotoxins might alter cytokine production and stimulate pro-inflammatory factors, which would then affect the fish immune system and finally promote tissue damage and apoptosis. Meanwhile, this assumption follows mainly from experimental studies using MCs as the model toxicant when findings regarding other cyanotoxins are strictly limited and correspondent mechanisms, which have to be the focus of further research.

## 7. Endocrine Disruption in Fish Induced by Cyanotoxins

After being acutely exposed to M. aeruginosa (4.4 × 10^5^, 7.2 × 10^5^, and 10.0 × 10^5^ cells/mL), both plasma sex hormone levels and the expression of genes implicated in the steroidogenesis pathway were drastically changed in female zebrafish [142]. Moreover, male zebrafish that were treated with 1, 5, 20 μg/L or 0.3, 1, 3, 10, and 30 μg/L MC-LR for 30 d showed a decrease in their gonad–somatic index. All exposed females revealed an increase in plasma 17-estradiol (E2), testosterone, 11-ketotestosterone, and follicle-stimulating hormone, whereas all treated male specimens had an alteration in plasma levels of testosterone, follicle-stimulating hormone, and luteinizing hormone, accompanied by extensive upregulation of steroidogenic genes, especially cyp19a, and downregulation of brain nuclear receptors (gr and mr) [143,144,145]. Similar results were presented not only in fish but also in murine models. For example, oral exposure to MC-LR (3.75–30 μg/kg bw per day) induced gonadotropin-releasing hormone expression in a dose- and duration-dependent manner and subsequently suppressed the expressions of testosterone, follicle-stimulating hormone, and luteinizing hormone in BALB/c mice [146]. Both MCs and crude extracts from MC-producing cyanobacteria strains can induce alterations in sex hormones and their ratio. In addition, some molecular events interplay with histomorphological evidence of retarded oogenesis and spermatogenesis in adult zebrafish [143] and, finally, are realized in organismal disorders, including reproductive dysfunction, such as lower fecundity rates, fertilization rates, and hatch success, as well as deformity, growth retardation, and organ malfunction in embryos and juveniles of zebrafish [147,148,149].

One of the most sensitive endpoints for measuring endocrine-disrupting chemical exposure is a change in vitellogenin (Vtg) levels in male fish. Vtg is a yolk precursor protein synthesized by the liver in response to estradiol stimulation and is essential for vitellogenesis, oocyte maturation, and yolk biosynthesis. Its gene is estrogen-regulated; hence its expression is normally suppressed in male fish. Conversely, xenoestrogen binding to male hepatic estrogen receptors can cause Vtg induction [150]. Although cyanotoxins have estrogenic potential, the literature on Vtg stimulation in fish is relatively ambiguous. On the one hand, it was reported that male larvae zebrafish exposed to lyophilized M. aeruginosa containing 4.5 μg/L MC-LR had higher expression of the Vtg gene (19.2-fold to >100-fold on arrays and 619-fold confirmed by quantitative PCR) [151]. On the other hand, long-term exposure of zebrafish to pure MC-LR in a concentration range of 1–1000 μg/L provoked no changes or even a considerably decreased change in Vtg expression and whole-body concentration in males, as reported by Rogers et al. [151] and Qiao et al. [152]. Reduced Vtg expression may be associated with the emergence of MC-LR-specific toxicity, seen at nanomolar concentrations [153], and subsequent liver lesions caused by the mentioned hepatotoxin. Meanwhile, the female reproductive system is vulnerable to the effects of MCs, in some cases, much more so than the male ones [152]. In particular, MCs were able to stimulate apoptotic processes in fish ovaries monitored by Bcl-2, BAX, and caspase 3 [152], dysregulate proteins involved in the development of gonads and ovogenesis [154], provoke prominent histological lesions in the gonads, decrease reproductive output monitored by vitellogenin and choriogenin expression, shortage the percentage of mature oocytes, number of spawned eggs, as well as alter plasma levels of 17β-estradiol, testosterone, and Vtg [142,155].

It is highly likely that the level of endocrine-disrupting activity of cyanotoxins depends on their structure and ability to bind to estrogen receptors. Using a yeast estrogen screen assay, CYN and ANA were shown to modulate the 17β-estradiol-induced estrogenic activity, and CYN was proven to be a much more potent endocrine disruptor than ATX-a [156]. Because CYN oxidation by the FeIII-B*/H_2_O_2_ system reshaped it and produced open-ring by-products, CYN’s binding affinity to estrogenic receptors was reduced, which resulted in lower endocrine-disrupting potential [156]. Similar experiments were carried out for MC-LR and NOD. It was shown that 17β-estradiol (at picomolar concentrations), NOD-R, and MC-LR (at nanomolar) induced luciferase activity [153]. In the case of low concentrations (<10 nM), in which no cytotoxicity of MC-LR was observed, the induction of the luciferase activity was significantly higher with MC-LR than NOD-R. Although cyanotoxins’ estrogenicity is lower than that of E2, it is much higher than that of the environmental estrogens bisphenol A (estradiol equivalence factor (EEF) = 2.5 × 10^−5^) and 4-nonylphenol (EEF = 1.25 × 10^−5^) obtained using luciferase reporter gene assays with MVLN cells [153,157]. As a result, the endocrine-disrupting potential of cyanotoxins is a major concern for wildlife, and the lack of studies in the field necessitates further research into the effects of cyanotoxins on low vertebrates, including fish.

MCs can promote reproductive toxicity through different mechanisms. From these, macroautophagy and endoplasmic reticulum stress are the most studied. Among the proteins involved in autophagy, soluble LC3, a key player in the later formation of autophagosomes, was found to increase at relatively low MC-LR concentrations [158]. Further, some studies showed that MC-LR could pave the way for the overexpression of endoplasmic reticulum stress and autophagy-related proteins, such as GRP78, ATF-6, PERK, IRE1, and CHOP, on the one hand, and Beclin1, ATG16, and ATG5-ATG12 on the other, as well as the formation of autophagic vesicles in germ cells [107,158], indicating that MCs may trigger autophagy and endoplasmic reticulum stress.

When the effect of MCs on the endocrine and reproductive systems has been the focus of studies, similar effects of other widespread cyanotoxins are still largely unknown. Treatment of the late vitellogenic staged (>0.58 mm diameter) follicles with 1000 μg/L CYN significantly increased germinal vesicle breakdown as an indication of oocyte maturation, Caspase-3 activity, and hCG-induced testosterone secretion. Further, CYN reduced the expression of 3βhsd, which might affect progesterone synthesis from pregnenolone and synthesis of 17α-hydroxyprogesterone from 17α-hydroxypregnenolone in the ovary and induce the aromatase cyp19a1, a key player in the biosynthesis of estrogens [159]. Following exposure to CYN, prominent histopathological alterations and a decrease in cell types from all three phases of spermatogenesis in zebrafish testis, as well as testosterone secretion and changes in fshr, lhr, and igf3 expression, were noted [160]. While the effects of MCs and CYN on fish cannot be directly compared due to a lack of evidence, it can be unambiguously indicated that exposure to both cyanotoxins can adversely affect fish reproduction. MCs and CYN can cause estrogenic effects and disturb the function of the endocrine system. Cyanotoxins alter serum and testicular hormone levels because of their effects on the neuroendocrine system, gonad injury, and interference with the expression of genes involved in sex hormone production.

## 8. Cyanotoxin Exposure Triggers Mitochondrial Damage and Endoplasmic Reticulum Stress in Fish

Due to their essential role in keeping cellular structures and processes running, mitochondria are easy targets for many different types of toxicants. When mitochondrial function is altered, the mitochondrial membrane and potential (MMP) are altered. The MMP is formed by a chemical and electrical gradient caused by the unequal distribution of protons and other ions on either side of the inner mitochondrial membrane. Functions of the mitochondria, such as oxidative phosphorylation and ATP production, need the MMP. It has been shown that a sub-chronic exposure of zebrafish to 20 μg/L provoked nucleus deformation and edematous mitochondria in the spleen [161]. Further, exposure of C. auratus lymphocytes to MC-RR (10 nmol/L) for up to 6 h caused a rapid disruption in mitochondrial membrane potential simultaneously, with massive calcium influx and reactive oxygen species production [162]. These findings were similar to the results observed in murine models. In particular, primary cultured rat hepatocytes were treated with moderate and high concentrations of MCs (equivalent to 12.5 and 125 µg lyophilized algae cells/mL, respectively) and showed a significant decrease in the fluorescence intensity of mitochondria, indicating the release of Rh-123 from mitochondria into the cytosol and a decrease in mitochondrial membrane potential, which appeared before the LDH leakage as the sign of hepatotoxicity [163,164]. The effects of MCs were linked to both MMP inhibition and the induction of the mitochondrial permeability transition. Membrane potential depolarization is most likely an early event in MC toxicity and acts as a downstream mediator and promoter of MC-induced apoptosis.

In recent years, several studies have uncovered numerous MC-induced apoptosis pathways in different model systems, including fish. MC-LR exposure ultimately results in the accumulation of cyanotoxin following the activation of cellular responses related to oxidative stress, apoptosis, DNA repair, and carcinogenicity [83]. It is known that apoptosis may be triggered by damaging the cytoskeleton as well as by targeting the mitochondria, endoplasmic reticulum, and epigenetic modification pathways [107]. The activation of calpain and Ca^2+/^calmodulin-dependent protein kinase by MCs, as well as the production of reactive oxygen species, may facilitate apoptosis [165]. Alternatively, MC-LR leads to the activation of the ER stress response in a wide range of organisms, including fish [34,166]. In zebrafish liver, MCs were able to induce the proven marker of ERS, GRP78, an endoplasmic reticulum chaperone and stabilizing agent of protein folding under endoplasmic reticulum stress [166]. The JNK protein is then activated by IRE1 and phosphorylates the Bcl2 protein, allowing for caspase-3 activation and the execution of apoptosis [167].

To damage the mitochondria, cyanotoxins, including MCs, CYN, and MC-produced cyanobacterial strains, cause an increase in intracellular ROS in fish that definitely has to act on the mitochondria’s outer membrane [54,100,102,105,106]. Based on the results from mammals, the abovementioned ROS induction may lead to a mitochondrial membrane potential drop and the opening of the mitochondrial permeability transition pore [168]. Following these events, cytochrome c is released through the mitochondrial permeability transition pore, which stimulates the activation of the downstream caspase family proteins [169]. In zebrafish, the mRNA expression of executor caspases 3a and 3b was increased and the anti-apoptotic regulator Bcl-2 expression was decreased after exposure to the extracts of non-CYN and non-MC-producing A. gracile and R. raciborskii of Central European origin or purified MC-LR [54]. In zebrafish, these proapoptotic events coincided with the transcriptional upregulation of GADD45 [54], proteins that have been implicated in the regulation of many cellular functions, such as DNA repair, cell cycle control, senescence, and genotoxic stress [170]. Not only MCs or cyanobacterial strains stimulate apoptosis in fish in close relation to ROS overproduction. Similar patterns have been noted for CYN and anatoxin [54,171]. For example, an increase in caspase-3 activity was observed simultaneously with an increase in lipid peroxidation in carp hepatocytes after a CYN analog containing guanidine, hydroxyl, and uracil functionalities [105]. Further, ATX-a can promote lymphocyte apoptosis in C. auratus after short-term acute in vitro exposure, observed by the number of apoptotic cells in close relation to significant intracellular oxidative stress and destroying the antioxidant system in lymphocytes [171]. To this end, all existing data clearly suggest that oxidative stress and its influence on mitochondria play important roles in cyanotoxin-induced apoptosis.

Shifts in cytosolic calcium content are known to initiate crucial cellular signaling pathways and metabolic activities. Apoptosis, along with other forms of cell death, is also under its strict control. It is widely recognized that an excess of Ca^2+^ in the cell may be extremely hazardous, causing huge activation of proteases and phospholipases and paving the way for mitochondrial swelling, which can ultimately end up in the loss of cell integrity and function followed by necrosis. In general, MCs were reported to affect Ca^2+^ channels in mammal cell models and stimulate a concentration-dependent rise in basal cytosolic Ca^2+^ [172]. In contrast, no clear pattern in cytosolic Ca^2+^ has been found in MCs or cyanobacterial extract-treated fish. On the one hand, it was shown that purified MC-LR had no effect on Ca^2+^ uptake in basolateral plasma membrane vesicles, endoplasmic reticulum, or mitochondria from tilapia gills, although extracts of both investigated M. aeruginosa strains (7820 and CYA 43) progressively inhibited Ca^2+^ uptake with increasing doses [173], which might make them more resistant to apoptosis [174]. On the other hand, more recent work has stressed that incubation of fish lymphocytes with MCs results in a rapid and significant elevation of intracellular Ca^2+^, which can be an early or primary event in MC-induced apoptosis [162]. Obviously, intracellular Ca^2+^ appears to have a crucial role in the initiation of the common pathways in programmed cell death and pretends to be an early apoptotic signal mediator; however, more mechanistic studies on fish are needed, which can shed light on cyanotoxins’ toxicity and the consequences of algal blooms on wildlife health status and biodiversity.

## 9. Conclusions and Perspectives

Over the past few decades, advances have been made in understanding the physiology, toxicity, and environmental dynamics of cyanobacteria and their metabolites. However, additional challenges have arisen, mainly due to global climate changes and increasing eutrophication, that push us to anticipate the scenario and repercussions of algal blooms in a warmer and more polluted future. Cyanotoxins, high concentrations of which can be found during algal blooms, can induce deleterious health effects and outcomes in wildlife and humans and cause biodiversity decline. To date, over 2000 cyanobacterial metabolites have been identified, and MCs are the most common kind of cyanotoxin found in aquatic environments across the world, which, therefore, determines the main points regarding the cyanobacterial bioactive metabolites’ toxicity discovered for microcystins. Approximately 400 papers that match the keywords “cylindrospermopsin” and “toxicity” were indexed in the PubMed database by the end of 2022, among which less than 60 were searchable using the combination of keywords “cylindrospermopsin”, “toxicity”, and “fish”. However, as they become more prevalent globally, other cyanotoxins (such as CYN, ATX-a, and STXs) and their effects on biota must be thoroughly investigated. These changes might have added value to the expanding toxicological database regarding cyanotoxins worldwide.

**Figure 1:**
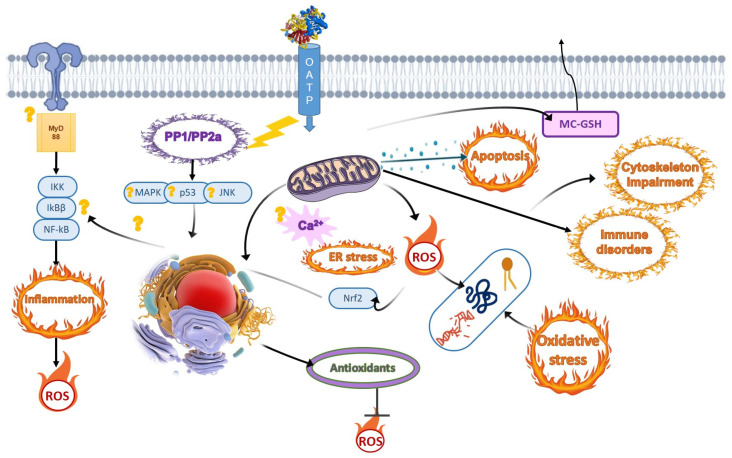
Schematic representation of the cellular and molecular events in fish induced by microcystins.

Cyanotoxin hazard stems from the fact that they can bioaccumulate in fish and subsequently be intrusively introduced into the trophic chain. Cyanotoxins on the molecular and cellular levels can cause a wide range of non-specific responses, including an imbalance in antioxidants, an accumulation of oxidative lesions, immunotoxicity, neuro-endocrine disorders, and geno- and cytotoxicity, in addition to damaging specific targets, such as protein phosphatases, in the case of MCs. These adverse outcomes manifest as oxidative stress, endoplasmic reticulum stress, mitochondrial dysfunction, inflammation, and apoptosis/autophagy (Figure 1). All these events at the organismal and populational levels pose a serious threat to fish populations, which can also affect humans that consume fish or water from the lake. Therefore, it is critical to continuously monitor the appearance of cyanobacterial strains and levels of various cyanotoxins they produce in surface waters worldwide, which may be the most effective way of ensuring the sustainability of water resources and the conservation of commercially important fish species.

## Data Availability

No original data were generated for this paper.
